# Association of serum s-adenosylmethionine, s-adenosylhomocysteine, and their ratio with the risk of dementia and death in a community

**DOI:** 10.1038/s41598-022-16242-y

**Published:** 2022-07-20

**Authors:** Akane Mihara, Tomoyuki Ohara, Jun Hata, Sanmei Chen, Takanori Honda, Sonam Tamrakar, Akiko Isa, Dongmei Wang, Kuniyoshi Shimizu, Yoshinori Katakura, Koji Yonemoto, Tomohiro Nakao, Takanari Kitazono, Toshiharu Ninomiya

**Affiliations:** 1 Department of Neuropsychiatry, Graduate School of Medical Sciences, Kyushu University, 3-1-1 Maidashi, Higashi-ku, Fukuoka, 812-8582 Japan; 2 Department of Epidemiology and Public Health, Graduate School of Medical Sciences, Kyushu University, Fukuoka, Japan; 3 Center for Cohort Studies, Graduate School of Medical Sciences, Kyushu University, Fukuoka, Japan; 4 Department of Medicine and Clinical Science, Graduate School of Medical Sciences, Kyushu University, Fukuoka, Japan; 5 Global Health Nursing, Department of Health Sciences, Graduate School of Biomedical and Health Sciences, Hiroshima University, Hiroshima, Japan; 6 Department of Agro-Environmental Sciences, Faculty of Agriculture, Kyusyu University, Fukuoka, Japan; 7 Department of Bioscience and Biotechnology, Faculty of Agriculture, Kyushu University, Fukuoka, Japan; 8 Division of Biostatistics, School of Health Sciences, Faculty of Medicine, University of the Ryukyus, Nishihara, Japan

**Keywords:** Dementia, Cognitive ageing, Biochemistry, Epidemiology

## Abstract

We examined the association of serum s-adenosylmethionine (SAM), s-adenosylhomocysteine (SAH) (methionine metabolites), and their ratio on the risk of dementia and death in a community-dwelling population of older Japanese individuals. 1371 residents of Hisayama, Japan, aged 65 years or older and without dementia, were followed for a median of 10.2 years (2007–2017). We divided serum SAM, SAH, and SAM/SAH ratio into quartiles. Cox proportional hazards models were used to estimate the hazard ratios (HRs) and their 95% confidence intervals (CIs) of serum SAM, SAH, and SAM/SAH ratio levels on the risk of a composite outcome of all-cause dementia or death, and each outcome. During the follow-up, 635 participants developed all-cause dementia and/or died, of which 379 participants developed dementia and 394 deaths occurred. The multivariable-adjusted HRs of the composite outcome decreased significantly with increasing serum SAM levels (*P* for trend = 0.01), while they increased significantly with higher serum SAH levels (*P* for trend = 0.03). Higher serum SAM/SAH ratio levels were significantly associated with a lower risk of the composite outcome (*P* for trend = 0.002), as well as with lower risk of each outcome. Our findings suggest that the balance of methionine metabolites may closely associate with the risk of dementia and death.

## Introduction

Methionine is an essential amino acid that is involved in protein synthesis and the regulation of protein function, and is metabolized in a complex manner by methionine metabolites, related enzymes, and the vitamin B group^1,2^. Methionine metabolism has been reported to be associated with regulation of aging and lifespan through antioxidative defense and various methylations^3–5^. In addition, a population-based prospective study showed that the risk of dementia decreased significantly with increases in the methionine/homocysteine ratio.^6^ These findings suggest that methionine metabolites may play an important role in the maintenance of cognitive function and prolongation of life expectancy.

S-adenosylmethionine (SAM), a product of methionine, is known as a key metabolite in a variety of methylation processes of DNA, RNA, and proteins^7^. Recently, some animal studies showed that elevated SAM was associated with cognitive improvement and lifespan extension^8–11^, while the elevation of s-adenosylhomocysteine (SAH), a metabolite obtained after removing a methyl group from SAM, was associated with lifespan suppression^12^. Small-scale clinical studies also showed that patients with Alzheimer’s disease (AD) had lower levels of the SAM/SAH ratio (another indicator of methylation status) in plasma or cerebrospinal fluid than those with normal cognition^13,14^. Therefore, we hypothesize that the levels of serum SAM, serum SAH, and the serum SAM/SAH ratio are associated with risk of dementia and death. However, no population-based prospective study has examined these associations comprehensively. Therefore, we aimed to elucidate the association between methionine metabolites, namely serum SAM, SAH, and SAM/SAH ratio, and the risk of dementia and death in a community-dwelling older Japanese population.

## Methods

### Study populations

The Hisayama Study is a population-based prospective longitudinal study of cerebrovascular diseases and dementia in the town of Hisayama, a suburban community adjacent to the city of Fukuoka in Japan^15,16^. Since 1961, we have performed surveys of the neurological and physical condition of residents every 1 to 2 years. Comprehensive surveys of dementia have also been carried out every 5 to 7 years since 1985 in the older residents. In 2007–2008, a total of 1581 residents aged ≥ 65 years (participation rate = 88.6%) underwent a screening survey. After the exclusion of 3 residents who refused to participate in this survey, 161 with prevalent dementia, and 46 without available data of serum SAM and SAH, the remaining 1371 residents (596 men and 775 women) were enrolled in the present study. This study was approved by the Kyushu University Institutional Review Board for Clinical Research, and all methods of this study were performed in accordance with Declaration of Helsinki and Ethical Guidelines for Medical and Biological Research Involving Human Subjects in Japan (https://www.mhlw.go.jp/content/000769923.pdf [Japanese]). We obtained written informed consent from all the participants.

### Follow-up survey of dementia and death

We followed up the participants from their baseline screening examination until the onset of dementia, death, or November 30, 2017 (median 10.2 years, interquartile range 9.3–10.4 years). Details of the follow-up survey on dementia were published previously^16^. In brief, information on new events of dementia and death was collected through a daily monitoring system established by the study team, local physicians, and members of the town’s Health Office. In this system, the physicians in the study team regularly visited clinics, hospitals, and the town’s office to collect information on events of dementia or stroke, including suspected cases. Regular health examinations, including physical and neurological examinations, were also repeated every year to obtain information on new events of dementia missed by the monitoring system. Health information was checked annually by letter or telephone for any individuals who did not undergo regular examination or who had moved away from town. When a participant was suspected of having new neurological symptoms, including dementia, he or she was carefully evaluated by an expert psychiatrist and expert stroke physician of the study team. In addition, comprehensive assessment of cognitive function, including neuropsychological tests, such as the Mini-Mental State Examination^17^, was conducted in 2012 and 2017 to detect dementia cases as accurately as possible. In addition, when a participant died, we collected and fully examined all the available medical information, including neuroimaging (CT/MRI), and interviewed the family and attending physician of the deceased. During the follow-up, 394 participants died and 247 of the decedents underwent an autopsy. No participants were lost to follow-up.

### Diagnosis of dementia

The diagnosis of dementia was made according to the guidelines of the Diagnostic and Statistical Manual of Mental Disorders, 3rd edition, revised^18^. The criteria of the National Institute of Neurological and Communicative Disorders and Stroke and the Alzheimer’s Disease and Related Disorders Association^19^ and the criteria of the National Institute of Neurological Disorders and Stroke-Association International pour la Recherche et l’Enseignement en Neurosciences were used to diagnose AD and vascular dementia (VaD)^20^, respectively. The diagnosis of probable or possible dementia subtypes was based on the clinical information and morphological analysis of neuroimages. For autopsied participants with dementia, we also made a diagnosis of definite dementia subtypes based on neuropathological and clinical information. The detailed procedure used to diagnose autopsied cases with dementia was reported previously^21^. The case of every participant suspected of having dementia and every deceased participant during the follow-up period was adjudicated based on all available medical information by expert psychiatrists and expert stroke physicians to confirm the absence or presence of dementia and its subtypes.

### Quantitation of serum SAM and serum SAH concentrations

Detailed information on the quantitation of serum SAM and serum SAH concentrations is provided in the Supplementary Methods. The levels of serum SAM, serum SAH, and the serum SAM/SAH ratio were divided into quartile categories (SAM: quartile 1 = 10.11–46.39 nmol/L, quartile 2 = 46.40–60.70 nmol/L, quartile 3 = 60.71–88.70 nmol/L, quartile 4 = 88.71–579.85 nmol/L; SAH: quartile 1 = 8.96–16.18 nmol/L, quartile 2 = 16.19–18.95 nmol/L, quartile 3 = 18.96–23.47 nmol/L, quartile 4 = 23.48–363.89 nmol/L; SAM/SAH ratio: quartile 1 = 0.33–2.32, quartile 2 = 2.33–3.06, quartile 3 = 3.07–4.52, quartile 4 = 4.53–15.96). We also measured serum methionine (Met) and serum total homocysteine (tHcy) concentrations. Detailed information on the quantitation of serum Met and serum tHcy concentrations is shown in the Supplementary Methods.

### Measurements of covariates

In the baseline survey, the information on lifestyle factors including educational status, smoking habits, alcohol intake, medical history, and use of anti-hypertensive agents, glucose-lowering agents, and lipid-modifying agents was collected by trained registered nurses using a self-administered questionnaire in a face-to-face survey for all participants, including those who had difficulty completing or who were unable to complete the questionnaire. We defined low education as ≤ 9 formal educational years. Smoking habits and alcohol intake were classified as either current habitual or not. Participants engaging in sports or other forms of exercise at least three times a week during their leisure time were included in the regular exercise group. Blood pressure was measured 3 times after > 5 min of rest in the sitting position, and the mean value of the 3 measurements was calculated. Hypertension was defined as systolic blood pressure ≥ 140 mmHg or diastolic blood pressure ≥ 90 mmHg or current use of antihypertensive agents. We measured plasma glucose levels by using the hexokinase method and determined diabetes as follows: fasting glucose ≥ 7.0 mmol/L, casual or 2-h 75 g oral glucose postloaded glucose level ≥ 11.1 mmol/L, and/or use of insulin or oral hypoglycemic agents. We also measured serum total cholesterol levels enzymatically. History of stroke was determined by using all clinical information of the Hisayama Study. We measured body height and weight in light clothing without shoes and calculated the body mass index (BMI) (kg/m^2^). Obesity was defined as BMI ≥ 25 kg/m^2^. We defined electrocardiogram abnormalities as atrial fibrillation (Minnesota Code 8–3), ST depression (4–1, 2, 3), or left ventricular hypertrophy (3–1). Use of vitamin B supplements was defined as taking vitamin B supplements containing any of B2, B6, B12, or folic acid. Serum alanine aminotransferase (ALT) levels were enzymatically measured in accordance with the consensus method of the Japan Society of Clinical Chemistry. Serum high-sensitivity C-reactive protein (hs-CRP) concentrations were measured using a modified version of the Behring Latex-Enhanced CRP assay on a Behring Nephelometer BN-100 (Behring Diagnostics, Westwood, MA).

### Statistical analysis

The trend in the age- and sex-adjusted mean values and the frequencies of baseline covariates according to serum SAM, serum SAH, and the serum SAM/SAH ratio levels were computed by using linear or logistic regression analysis, respectively. The incidence rate of dementia and mortality rate were calculated with the person-year method. The hazard ratios (HRs) and their 95% confidence intervals (CIs) for the development of outcomes—namely, the composite outcome of all-cause dementia and death (hereafter “all-cause dementia or death”), all-cause dementia, all-cause death, and dementia subtypes for each quartile of serum SAM, serum SAH, serum SAM/SAH ratio, serum Met, serum tHcy, and serum Met/tHcy ratio were estimated by using a Cox proportional hazards model^22^. The risk of outcomes of interest per 1-standard deviation (SD) increment in continuous values of serum SAM, SAH, and SAM/SAH ratio were also estimated, where these values were log-transformed before fitting the model because their distributions were skewed to the right (Supplementary Fig. S1). We also assessed the association between serum SAM, serum SAH, and the serum SAM/SAH ratio and risk of all-cause dementia or death stratified by the levels of serum tHcy. The heterogeneity in the association between subgroups was examined by adding multiplicative interaction terms to the relevant Cox model.

In addition, we used restricted cubic splines^23^ to visually assess the shape of the association between the serum SAM/SAH ratio levels and the risk of dementia or death whether or not there were inappropriate linearity assumptions. Five knots were placed on the x-axis at the 5th, 25th, 50th, 75th, and 95th percentiles of the serum SAM/SAH ratio levels (1.33, 2.33, 3.07, 4.53, and 7.78, respectively), and the 5th percentile (1.33) was set as the reference value. The y-axis shows the multivariable-adjusted HR. All statistical analyses were performed with SAS, version 9.4 (SAS Institute, Cary, NC). Two-sided values of *P* < 0.05 were considered statistically significant.

## Results

The median values of serum SAM, SAH and SAM/SAH ratio levels at baseline were 60.71 nmol/L (interquartile range 46.40–88.71), 18.96 nmol/L (interquartile range 16.19–23.48), and 3.07 (interquartile range 2.33–4.53), respectively (Supplementary Fig. S1). The age- and sex-adjusted baseline characteristics of the study population according to serum SAM, SAH, and SAM/SAH ratio levels are summarized in Table 1 and Supplementary Tables S1–3. The mean value of BMI, the frequencies of low education, use of anti-hypertensive agents, hypertension, use of lipid-modifying agents, obesity, diabetes mellitus, use of glucose-lowering agents, and electrocardiogram abnormalities, and the geometric means of serum ALT increased significantly with higher levels of serum SAM, while the mean value of serum total cholesterol decreased significantly with higher levels of serum SAM (Table 1 and Supplementary Table S1). In regard to serum SAH levels (Table 1 and Supplementary Table S2), the mean values of age, systolic blood pressure, diastolic blood pressure and BMI, the frequencies of men, low education, use of anti-hypertensive agents, hypertension, obesity, diabetes mellitus, use of antidiabetic medication, electrocardiogram abnormalities, and history of stroke, and the geometric means of serum ALT and serum hs-CRP increased significantly with higher levels of serum SAH, while the mean value of serum total cholesterol decreased significantly with higher levels of serum SAH. With respect to the serum SAM/SAH ratio levels, the mean value of age and the frequencies of men and history of stroke decreased significantly with higher levels of the serum SAM/SAH ratio, while the geometric means of serum ALT increased significantly with higher levels of the serum SAM/SAH ratio (Table 1 and Supplementary Table S3).

**Table 1:** Age- and sex-adjusted baseline characteristics of participants according to serum SAM, SAH and SAM/SAH ratio levels (2007).

Risk factors	Serum SAM (nmol/L)			Serum SAH (nmol/L)			Serum SAM/SAH ratio		
	Q1	Q4	*P* for trend ^a^	Q1	Q4	*P* for trend ^a^	Q1	Q4	*P* for trend ^a^
	(10.11–46.39)	(88.71–579.85)		(8.96–16.18)	(23.48–363.89)		(0.33–2.32)	(4.53–15.96)	
	(n = 342)	(n = 343)		(n = 341)	(n = 343)		(n = 340)	(n = 344)	
Age, mean (SD), years	74 (7)	74 (7)	0.94	72 (7)	77 (7)	< 0.001	76 (7)	73 (7)	< 0.001
Male, %	41.4	44.7	0.61	19.4	67.6	< 0.001	57.6	34.5	< 0.001
Education ≤ 9 years, %	42.6	51.0	0.04	44.2	56.5	0.002	50.7	50.3	0.77
Systolic blood pressure, mean (SD), mmHg	135 (18)	135 (18)	0.53	133 (19)	137 (19)	0.01	135 (18)	135 (18)	0.71
Diastolic blood pressure, mean (SD), mmHg	79 (10)	79 (10)	0.69	78 (10)	80 (10)	0.001	79 (10)	78 (10)	0.12
Use of anti-hypertensive agents, %	42.9	58.4	< 0.001	38.0	60.8	< 0.001	48.8	50.0	0.63
Hypertension, %	61.2	71.0	0.005	54.4	76.5	< 0.001	65.2	64.1	0.92
Serum total cholesterol, mean (SD), mmol/L	5.4 (0.7)	5.2 (0.7)	0.01	5.4 (0.9)	5.2 (0.9)	0.001	5.2 (0.9)	5.3 (0.7)	0.88
Use of lipid-modifying agents, %	19.2	27.2	0.02	24.5	23.9	0.47	20.9	24.6	0.12
Body mass index, mean (SD), kg/m^2^	22.3 (3.3)	23.5 (3.3)	< 0.001	22.3 (3.5)	23.9 (3.5)	< 0.001	22.8 (3.3)	23.1 (3.3)	0.16
Obesity, %	20.0	32.0	< 0.001	16.6	38.1	< 0.001	24.8	25.9	0.85
Diabetes mellitus, %	15.8	29.1	< 0.001	20.1	29.6	0.004	18.5	23.9	0.09
Use of glucose-lowering agents, %	7.8	14.1	0.01	9.6	18.1	< 0.001	10.8	11.0	0.80
Electrocardiogram abnormalities ^b^, %	16.2	24.2	0.02	16.7	24.1	0.01	20.8	21.4	0.88
History of stroke, %	4.7	4.9	0.92	3.6	9.7	0.004	7.7	4.2	0.046
Smoking habits, %	8.0	8.3	0.91	6.3	9.2	0.12	8.5	7.5	0.84
Alcohol intakes, %	34.4	36.4	0.79	38.3	34.8	0.33	30.7	37.4	0.20
Regular exercise ^c^, %	12.7	16.3	0.15	14.6	11.1	0.28	12.1	16.7	0.09
Use of vitamin B supplements, %	5.5	6.8	0.65	6.0	6.8	0.56	5.3	5.8	0.63
Serum alanine transaminase, geometric means (95%CI), IU/L	16.4 (15.6–17.1)	19.1 (18.2–19.9)	< 0.001	16.3 (15.5–17.1)	19.3 (18.5–20.5)	< 0.001	16.9 (16.3–17.8)	18.2 (17.3–18.9)	0.02
Serum high-sensitivity C-reactive protein, geometric means (95%CI), mg/L	0.50 (0.44–0.58)	0.63 (0.55–0.71)	0.06	0.42 (0.37–0.48)	0.80 (0.70–0.92)	< 0.001	0.61 (0.54–0.70)	0.53 (0.46–0.60)	0.09

*CI* confidence interval, *SAH* s-adenosylhomocysteine, *SAM* s-adenosylmethionine, *SD* standard deviation.

^a^This table shows the mean values or frequencies of risk factors only for the Q1 and Q4 groups among the quartiles of serum SAM, SAH and SAM/SAH ratio levels due to the size of the table. Trends of each value were tested across all quartile groups by using the linear or logistic regression model. The values for all quartile groups are shown in the Supplementary Table S1 for serum SAM levels, the Supplementary Table S2 for serum SAH levels and the Supplementary Table S3 for serum SAM/SAH ratio levels.

^b^Electrocardiogram abnormalities were defined as left ventricular hypertrophy (Minnesota Code 3–1), ST depression (4–1, 2, 3), or atrial fibrillation (8–3).

^c^Regular exercise was defined as engaging in sports or other forms of exercise at least three times a week during leisure time.

During the 10-year follow-up period, 635 participants developed all-cause dementia or death (289 males and 346 females), of whom 379 participants developed all-cause dementia and 394 participants died. Among the incident dementia cases, 355 participants in all (93.7%) underwent some kind of morphological examination: 226 were evaluated by brain imaging, 13 underwent brain autopsy and 116 received both procedures. With regard to the dementia subtypes, 276 participants developed AD, 71 developed VaD, and 71 developed other subtypes of dementia; among them, 39 cases with a mixed type (25 cases were a mixed type of AD and VaD) were treated as events of the respective subtype in the analysis for that subtype.

Table 2 shows the adjusted HRs of all-cause dementia or death according to the quartile levels of serum SAM, SAH, and SAM/SAH ratio. The age- and sex-adjusted HRs of all-cause dementia or death decreased significantly with higher quartile levels of serum SAM (*P* for trend = 0.02), while they increased significantly with higher quartile levels of serum SAH (*P* for trend = 0.003). Moreover, higher serum SAM/SAH ratio levels were significantly associated with lower risk of all-cause dementia or death (*P* for trend < 0.001). These significant associations were unchanged even after adjustment for age, sex, education, hypertension, diabetes mellitus, serum total cholesterol, BMI, history of stroke, current smoking, current drinking, regular exercise and use of vitamin B supplements: the HRs (95% CIs) of all-cause dementia or death in the highest quartile level against the lowest were 0.76 (0.61–0.96) for serum SAM, 1.30 (1.02–1.66) for serum SAH, and 0.66 (0.53–0.83) for the serum SAM/SAH ratio, respectively. In addition, the multivariable-adjusted HRs (95% CIs) of all-cause dementia or death per 1-SD increment in the natural log-transformed serum SAM, serum SAH, and serum SAM/SAH ratio were 0.90 (0.83–0.97), 1.15 (1.06–1.25), and 0.88 (0.81–0.95), respectively. These associations were not altered substantially after additionally adjusting for serum ALT levels or serum hs-CRP levels (Supplementary Table S4). In the sensitivity analyses excluding incident all-cause dementia cases or deceased cases within the first 2 years of follow-up, there were significant inverse associations of serum SAM and the serum SAM/SAH ratio with the risk of a composite outcome of all-cause dementia or death (Supplementary Table S5).

**Table 2:** The risk of all-cause dementia or death according to serum SAM, SAH and SAM/SAH ratio levels (2007–2017).

	Persons at risk	No. of events	Crude incidence (per 1000 PYs)	Hazard ratio (95% CI)	
				Age- and sex-adjusted	Multivariable-adjusted^b^
**Serum SAM**					
Q1 (10.11–46.39)	342	161	60.7	1.00 (reference)	1.00 (reference)
Q2 (46.40–60.70)	343	161	59.4	0.88 (0.71–1.10)	0.83 (0.66–1.04)
Q3 (60.71–88.70)	343	159	58.4	0.80 (0.64–0.999)*	0.74 (0.59–0.93)*
Q4 (88.71–579.85)	343	154	54.6	0.79 (0.63–0.98)*	0.76 (0.61–0.96)*
*P* for trend				0.02	0.01
Per 1-SD increment^a^				0.91 (0.84–0.98)^$^	0.90 (0.83–0.97)^$^
**Serum SAH**					
Q1 (8.96–16.18)	341	127	43.0	1.00 (reference)	1.00 (reference)
Q2 (16.19–18.95)	344	145	50.7	1.02 (0.80–1.29)	0.99 (0.78–1.26)
Q3 (18.96–23.47)	343	153	55.0	1.01 (0.80–1.29)	0.98 (0.77–1.26)
Q4 (23.48–363.89)	343	210	91.0	1.42 (1.12–1.80)**	1.30 (1.02–1.66)*
*P* for trend				0.003	0.03
Per 1-SD increment^a^				1.17 (1.08–1.26)^$^	1.15 (1.06–1.25)^$^
**Serum SAM/SAH ratio**					
Q1 (0.33–2.32)	340	192	82.1	1.00 (reference)	1.00 (reference)
Q2 (2.33–3.06)	343	152	54.0	0.71 (0.57–0.88)**	0.73 (0.58–0.91)**
Q3 (3.07–4.52)	344	156	55.8	0.77 (0.62–0.95)*	0.79 (0.63–0.98)*
Q4 (4.53–15.96)	344	135	45.6	0.62 (0.50–0.78)**	0.66 (0.53–0.83)**
*P* for trend				< 0.001	0.002
Per 1-SD increment^a^				0.85 (0.78–0.92)^$^	0.88 (0.81–0.95)^$^

*CI* confidence interval, *PYs* person-years, *SAH* s-adenosylhomocysteine, *SAM* s-adenosylmethionine, *SD* standard deviation.

^a^1-SDs of natural log-transformed values of serum SAM, serum SAH, and the serum SAM/SAH ratio were 0.46, 0.32, and 0.54.

^b^Adjusted for age, sex, education, hypertension, diabetes mellitus, serum total cholesterol, body mass index, history of stroke, current smoking, current drinking, regular exercise and use of vitamin B supplements.

*P < 0.05, **P < 0.01 versus Q1.

^$^*P* < 0.05 per 1-SD increment.

**Figure 1:**
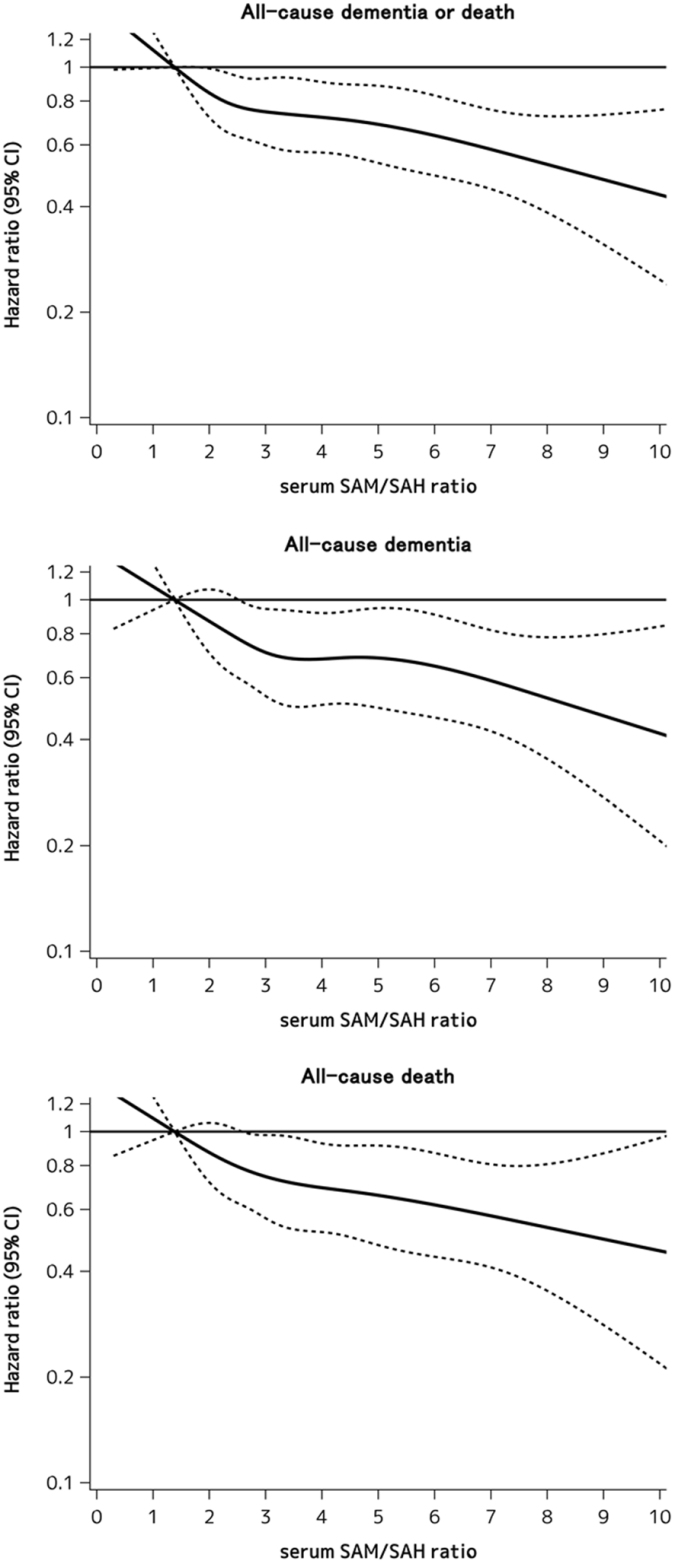
Restricted cubic spline for the association between the serum SAM/SAH ratio and risk of all-cause dementia or death. Solid lines represent the hazard ratio, and dashed lines represent the 95% CI of the hazard ratio. Knots were placed at the 5th, 25th, 50th, 75th and 95th percentiles (1.33, 2.33, 3.07, 4.53, and 7.78, respectively) of the serum SAM/SAH ratio. A reference point was set at the 5th percentile (1.33). The risk estimates were adjusted for age, sex, education, hypertension, diabetes mellitus, serum total cholesterol, body mass index, history of stroke, current smoking, regular exercise and use of vitamin B supplements. SAM, s-adenosylmethionine; SAH, s-adenosylhomocysteine; CI, confidence intervals.

We also estimated the adjusted HRs of the serum Met, tHcy, and Met/tHcy ratio levels on the risk of all-cause dementia or death, with division of the levels of serum Met, serum tHcy, and the serum Met/tHcy ratio into quartile categories (Supplementary Table S6). The results showed that there was no significant association between serum Met and Met/tHcy ratio levels and risk of all-cause dementia or death, while the multivariable-adjusted HR of all-cause dementia or death increased significantly with higher levels of serum tHcy (*P* for trend = 0.0495). Furthermore, we performed a subgroup analysis by serum tHcy levels, in which the cut-off value of serum tHcy was defined as 11.99 μmol/L (the fourth quartile vs. others). As shown in Fig. 1, the association between higher serum SAM and SAM/SAH ratio levels and lower risk of all-cause dementia or death was substantially unchanged regardless of the serum tHcy levels without any heterogeneities.

We also investigated the risk of developing all-cause dementia and the risk of all-cause death separately according to serum SAM, SAH, and SAM/SAH ratio levels. The risk of all-cause dementia decreased significantly with higher quartile levels of serum SAM (*P* for trend = 0.02) and the serum SAM/SAH ratio (*P* for trend = 0.01) after adjusting for the above-mentioned confounding factors, while no significant associations between serum SAH levels and risk of all-cause dementia were observed (Table 3). With regard to subtypes of dementia, the multivariable-adjusted risk of developing AD decreased significantly with higher quartile levels of serum SAM (*P* for trend = 0.04), and tended to decrease with higher quartile levels of the serum SAM/SAH ratio (*P* for trend = 0.09). On the other hand, there was no significant association between serum SAM, SAH, SAM/SAH ratio levels and the risk of developing VaD (Supplementary Table S7). In regard to all-cause death, higher quartile levels of serum SAH, but not SAM, were significantly associated with greater risk of all-cause death after adjusting for confounding factors (*P* for trend < 0.001). Meanwhile, there was a negative significant association between serum SAM/SAH ratio levels and the risk of all-cause death (*P* for trend = 0.001) (Table 4).

**Table 3:** The risk of all-cause dementia according to serum SAM, SAH and SAM/SAH ratio levels (2007–2017).

	Persons at risk	No. of events	Crude incidence (per 1000 PYs)	Hazard ratio (95% CI)	
				Age- and sex-adjusted	Multivariable-adjusted^b^
**Serum SAM**					
Q1 (10.11–46.39)	342	97	36.5	1.00 (reference)	1.00 (reference)
Q2 (46.40–60.70)	343	98	36.2	0.91 (0.69–1.21)	0.87 (0.66–1.16)
Q3 (60.71–88.70)	343	100	36.7	0.83 (0.63–1.10)	0.78 (0.58–1.04)
Q4 (88.71–579.85)	343	84	29.8	0.73 (0.54–0.97)*	0.70 (0.52–0.95)*
*P* for trend				0.03	0.02
Per 1-SD increment^a^				0.85 (0.77–0.95)^$^	0.85 (0.76–0.94)^$^
**Serum SAH**					
Q1 (8.96–16.18)	341	91	30.8	1.00 (reference)	1.00 (reference)
Q2 (16.19–18.95)	344	85	29.7	0.88 (0.65–1.18)	0.84 (0.62–1.14)
Q3 (18.96–23.47)	343	91	32.7	0.93 (0.69–1.26)	0.91 (0.67–1.23)
Q4 (23.48–363.89)	343	112	48.6	1.21 (0.90–1.63)	1.11 (0.81–1.50)
*P* for trend				0.17	0.41
Per 1-SD increment^a^				1.08 (0.97–1.21)	1.07 (0.96–1.20)
**Serum SAM/SAH ratio**					
Q1 (0.33–2.32)	340	112	47.9	1.00 (reference)	1.00 (reference)
Q2 (2.33–3.06)	343	87	30.9	0.66 (0.49–0.87)**	0.67 (0.50–0.89)**
Q3 (3.07–4.52)	344	93	33.2	0.72 (0.55–0.96)*	0.73 (0.55–0.97)*
Q4 (4.53–15.96)	344	87	29.4	0.63 (0.48–0.84)**	0.66 (0.50–0.88)**
*P* for trend				0.006	0.01
Per 1-SD increment^a^				0.84 (0.76–0.93)^$^	0.84 (0.76–0.93)^$^

*CI* confidence interval, *PYs* person-years, *SAH* s-adenosylhomocysteine, *SAM* s-adenosylmethionine, *SD* standard deviation.

^a^1-SDs of natural log-transformed values of serum SAM, serum SAH, and the serum SAM/SAH ratio were 0.46, 0.32, and 0.54.

^b^Adjusted for age, sex, education, hypertension, diabetes mellitus, serum total cholesterol, body mass index, history of stroke, current smoking, current drinking, regular exercise and use of vitamin B supplements.

*P < 0.05, **P < 0.01 versus Q1.

^$^*P* < 0.05 per 1-SD increment.

**Table 4:** The risk of all-cause death according to serum SAM, SAH and SAM/SAH ratio levels (2007–2017).

	Persons at risk	No. of events	Crude incidence (per 1000 PYs)	Hazard ratio (95% CI)	
				Age- and sex-adjusted	Multivariable-adjusted^b^
**Serum SAM**					
Q1 (10.11–46.39)	342	103	33.7	1.00 (reference)	1.00 (reference)
Q2 (46.40–60.70)	343	98	31.6	0.88 (0.66–1.15)	0.80 (0.60–1.06)
Q3 (60.71–88.70)	343	97	31.2	0.82 (0.62–1.08)	0.76 (0.57–1.01)
Q4 (88.71–579.85)	343	96	30.4	0.85 (0.64–1.12)	0.85 (0.64–1.14)
*P* for trend				0.20	0.27
Per 1-SD increment^a^				0.97 (0.88–1.07)	0.97 (0.87–1.08)
**Serum SAH**					
Q1 (8.96–16.18)	341	62	18.7	1.00 (reference)	1.00 (reference)
Q2 (16.19–18.95)	344	81	25.3	1.09 (0.78–1.52)	1.06 (0.76–1.49)
Q3 (18.96–23.47)	343	93	29.6	1.10 (0.79–1.53)	1.06 (0.76–1.48)
Q4 (23.48–363.89)	343	158	57.2	1.75 (1.28–2.39)**	1.65 (1.20–2.28)**
*P* for trend				< 0.001	< 0.001
Per 1-SD increment^a^				1.28 (1.18–1.39)^$^	1.29 (1.18–1.41)^$^
**Serum SAM/SAH ratio**					
Q1 (0.33–2.32)	340	141	50.2	1.00 (reference)	1.00 (reference)
Q2 (2.33–3.06)	343	89	28.4	0.67 (0.51–0.88)**	0.66 (0.50–0.88)**
Q3 (3.07–4.52)	344	92	29.3	0.76 (0.58–0.99)*	0.76 (0.57–0.99)*
Q4 (4.53–15.96)	344	72	21.6	0.55 (0.41–0.74)**	0.58 (0.43–0.78)**
*P* for trend				< 0.001	0.001
Per 1-SD increment^a^				0.83 (0.76–0.92)^$^	0.84 (0.76–0.93)^$^

*CI* confidence interval, *PYs* person-years, *SAH* s-adenosylhomocysteine, *SAM* s-adenosylmethionine, *SD* standard deviation.

^a^1-SDs of natural log-transformed values of serum SAM, serum SAH, and the serum SAM/SAH ratio were 0.46, 0.32, and 0.54.

^b^Adjusted for age, sex, education, hypertension, diabetes mellitus, serum total cholesterol, body mass index, history of stroke, current smoking, current drinking, regular exercise and use of vitamin B supplements.

*P < 0.05, **P < 0.01 versus Q1.

^$^*P* < 0.05 per 1-SD increment.

**Figure 2:**
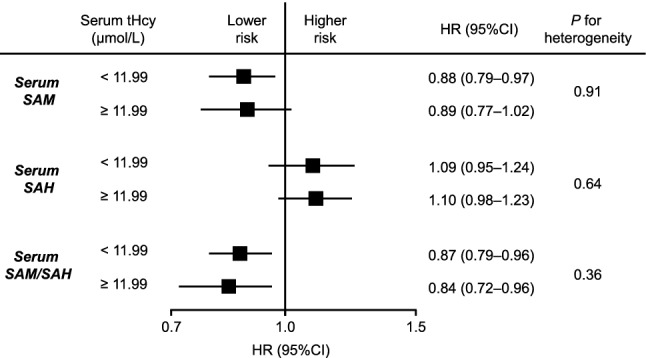
Association between serum SAM, SAH, and SAM/SAH ratio levels and all-cause dementia or death stratified by the levels of serum tHcy. HRs (95% CIs) of all-cause dementia or death were estimated per 1SD of the natural log transformed values of serum SAM, serum SAH, and the serum SAM/SAH ratio, respectively. The risk estimates were adjusted for age, sex, education, hypertension, diabetes mellitus, serum total cholesterol, body mass index, history of stroke, current smoking, regular exercise and use of vitamin B supplements. SAM, s-adenosylmethionine; SAH, s-adenosylhomocysteine; tHcy, total homocysteine; HR, hazard ratio; CI, confidence interval; SD, standard deviation.

Figure 2 shows the association between serum SAM/SAH ratio levels and the risk of the development of all-cause dementia or death, all-cause dementia and all-cause death, which were estimated by using the restricted cubic spline analyses. The risk of all-cause dementia or death decreased approximately linearly with higher levels of the serum SAM/SAH ratio. A similar linear shape of the association was also observed for the separate risks of all-cause dementia and all-cause death.

## Discussion

The present study demonstrated that the risk of all-cause dementia or death decreased significantly with elevating serum SAM/SAH ratio levels based on 10-year prospective data from a community-dwelling older Japanese population. In addition, higher serum SAM/SAH ratio levels were also significantly associated with lower risk of both all-cause dementia and all-cause death. In the present study, the risk of all-cause dementia or death decreased significantly with higher serum SAM level and increased significantly with higher serum SAH. The serum tHcy level has been reported to be associated with risk of all-cause mortality or dementia^24–27^. The present study also found that the risk of all-cause dementia or death increased significantly with higher levels of serum tHcy. However, the subgroup analysis of serum tHcy levels showed no heterogeneities in the association of higher serum SAM, SAH, and SAM/SAH ratio levels with lower risk of all-cause dementia or death between serum tHcy levels, possibly suggesting that the association of serum SAM, SAH, and SAM/SAH ratio levels with the risk of all-cause dementia or death was independent of serum tHcy levels. Therefore, these findings suggest that serum SAM and serum SAH play opposing roles on the development of dementia or death, and that there are close associations between the balance of methionine metabolites and risk of dementia and death.

As far as we know, no community-based prospective study has addressed the associations between serum SAM, SAH, and SAM/SAH ratio levels and risk of dementia and death. Some small clinical studies of fewer than 200 participants have compared plasma or cerebrospinal concentrations of SAM, SAH, and SAM/SAH ratio between AD patients and participants with normal cognition^13,14^. These studies have shown some similar findings—namely, that AD patients had significantly higher plasma and cerebrospinal SAH levels and lower SAM/SAH ratio levels than those with normal cognition—but the findings regarding the differences in plasma or cerebrospinal SAM levels between groups were inconsistent^13,14^. A population-based prospective study in Sweden showed that increased levels of the serum methionine/homocysteine ratio were significantly associated with lower risk of dementia^6^. In addition, a hospital-based prospective study examined the association between plasma SAM, SAH, and SAM/SAH ratio and risk of death and showed that the risk of death increased significantly with higher levels of plasma SAH and lower levels of plasma SAM and SAM/SAH ratio^28^. Our findings on the association of serum SAH and the serum SAM/SAH ratio with risk of all-cause death were consistent with the findings of this previous study. Taken together, these results suggest that there may be close associations between serum SAM, serum SAH, and the serum SAM/SAH ratio and the risk of dementia and death.

In the present study, higher levels of serum SAM were significantly associated with lower risk of all-cause dementia and AD. In the methionine metabolism, SAM is known to be a key metabolite involved in methylation homeostasis^7^. SAM has been reported to be associated with the suppression of *PSEN-1* and *BACE-1* gene expression related to the accumulation of amyloid β protein^29–32^, the activation of protein phosphatase 2A related to dephosphorylation of phosphorylated tau^8,33^, and the synthesis of glutathione related to antioxidative defense^34,35^. These findings suggest that SAM may play an important role in the protection of brain aging through maintenance of methylation homeostasis and reduction of oxidative stress. The findings from several animal studies that taking a SAM-supplemented diet significantly improved cognitive function support this hypothesis^8–10^. Meanwhile, the present study demonstrated that higher levels of serum SAH were significantly associated with increased risk of death. A possible mechanism underlying the association between serum SAH levels and death may be an acceleration of atherosclerosis. SAH has been reported to promote atherosclerosis by inducing endothelial cell dysfunction^36–38^ and proliferation and migration of vascular smooth muscle cells^39^. In the present study, because the incidence rate of VaD increased significantly with higher levels of serum SAH, our results may support this hypothesis.

The present study also found that higher levels of the serum SAM/SAH ratio were significantly associated with lower risk of dementia and death. Higher levels of the serum SAM/SAH ratio represent increased levels of production of SAM and clearance of SAH, and increased SAM production and SAH clearance may in turn improve cognitive function and extend lifespan through the suppression of oxidative stress, accumulation of amyloid-β and phosphorylated tau, and vascular damages. An experimental study using *Drosophila melanogaster* reported that suppression of SAH accumulation was associated with lifespan extension, and in the present study we discussed that a balance of methionine metabolites, especially clearance of the metabolic pathway between SAM and SAH, may be important for lifespan extension^12^. In addition, the plasma SAM/SAH ratio was reported to be significantly correlated with the intracellular SAM/SAH ratio in lymphocytes^40^, indicating that the levels of SAM, SAH and the SAM/SAH ratio in serum may reflect the corresponding levels in cells. Taken together, the present findings suggest that not only the concentrations of serum SAM and SAH but also the balance of methionine metabolites (serum SAM/SAH ratio) are important in the association of methionine metabolism with the risk of dementia and death.

The strengths of this study were the longitudinal population-based prospective design, the perfect follow-up of participants, and the ascertainment and diagnosis of dementia cases and their subtypes based on the detailed neuropsychological data, medical records, neuroimaging and autopsy. However, the present study had some limitations. First, our findings were based on a single measurement of serum SAM and SAH concentrations at baseline, which likely caused some misclassification in their levels. Second, we were unable to exclude the possibility that participants with prodromal dementia were included in the groups with lower levels of serum SAM and SAM/SAH ratio at baseline. In the sensitivity analyses after excluding dementia cases or death occurring within the first 2 years of follow-up, however, the significant inverse associations of serum SAM and the serum SAM/SAH ratio with the risk of all-cause dementia or death were still observed (Supplementary Table S5). Third, we could not perform detailed analysis regarding the subtypes of dementia and the cause of death because of the limited sample size. Fourth, the generalizability of the present findings to other ethnicities may be limited. Finally, we cannot rule out residual confounding by unmeasured confounders (e.g., dietary intake of methionine or the vitamin B group).

The present study demonstrated that higher levels of the serum SAM/SAH ratio were significantly associated with lower risk of a composite outcome of all-cause dementia and all-cause death, as well as with a lower risk of either outcome individually, in a general older Japanese population. These findings indicate the importance of the balance of methionine metabolites in the association of methionine metabolism with the risk of dementia and death. Further epidemiological and experimental studies are required to validate the findings of this study and to elucidate the mechanism underlying the association between methionine metabolism and the development of dementia and death.

## Supplementary Information

Supplementary Information.

## Data Availability

The datasets generated and analyzed in the present study are not publicly available because they contain confidential clinical and demographic data of the study participants. However, further information about the datasets is available with the permission of the principal investigator of the Hisayama Study (Toshiharu Ninomiya) on reasonable request for purposes of replicating procedures and results.
