# Supplementary material for: Association of serum s-adenosylmethionine, s-adenosylhomocysteine, and their ratio with the risk of dementia and death in a community
Source: Sci Rep. 2022 Jul 20;12:12427. doi: 10.1038/s41598-022-16242-y (PMC9300687; doi:10.1038/s41598-022-16242-y)
Supplement: Supplementary file 1 — Supplementary Information. [file 41598_2022_16242_MOESM1_ESM.docx]

| **Supplementary Table S1. Age- and sex-adjusted baseline characteristics of participants according to serum SAM levels (2007)** | | | | | |
| --- | --- | --- | --- | --- | --- |
| Risk factors | Serum SAM levels (nmol/L) | | | |  |
|  | Q1  (10.11–46.39) | Q2  (46.40–60.70) | Q3  (60.71–88.70) | Q4  (88.71–579.85) | *P* for trend |
|  | (n = 342) | (n = 343) | (n = 343) | (n = 343) |  |
| Age, mean (SD), years | 74 (7) | 74 (7) | 75 (7) | 74 (7) | 0.94 |
| Men, % | 41.4 | 45.8 | 41.9 | 44.7 | 0.61 |
| Education ≤ 9 years, % | 42.6 | 51.2 | 51.8 | 51.0 | 0.04 |
| Systolic blood pressure, mean (SD), mmHg | 135 (18) | 137 (18) | 138 (18) | 135 (18) | 0.53 |
| Diastolic blood pressure, mean (SD), mmHg | 79 (10) | 80 (10) | 80 (10) | 79 (10) | 0.69 |
| Use of anti-hypertensive agents, % | 42.9 | 45.9 | 47.4 | 58.4 | < 0.001 |
| Hypertension, % | 61.2 | 64.8 | 68.1 | 71.0 | 0.005 |
| Serum total cholesterol, mean (SD), mmol/L | 5.4 (0.7) | 5.3 (0.7) | 5.2 (0.7) | 5.2 (0.7) | 0.01 |
| Use of lipid-modifying agents, % | 19.2 | 23.7 | 23.8 | 27.2 | 0.02 |
| Body mass index, mean (SD), kg/m^2^ | 22.3 (3.3) | 22.8 (3.3) | 23.2 (3.3) | 23.5 (3.3) | < 0.001 |
| Obesity, % | 20.0 | 23.6 | 27.1 | 32.0 | < 0.001 |
| Diabetes mellitus, % | 15.8 | 20.3 | 20.3 | 29.1 | < 0.001 |
| Use of antidiabetic medication, % | 7.8 | 10.8 | 11.9 | 14.1 | 0.01 |
| Electrocardiogram abnormalities, % ^a)^ | 16.2 | 23.0 | 21.2 | 24.2 | 0.02 |
| History of stroke, % | 4.7 | 6.4 | 5.3 | 4.9 | 0.92 |
| Smoking habits, % | 8.0 | 8.6 | 8.5 | 8.3 | 0.91 |
| Alcohol intakes, % | 34.4 | 38.5 | 35.9 | 36.4 | 0.79 |
| Regular exercise, % ^b)^ | 12.7 | 12.3 | 13.4 | 16.3 | 0.15 |
| Use of vitamin B supplements, % | 5.5 | 7.8 | 6.5 | 6.8 | 0.65 |
| Serum alanine transaminase,  geometric means (95%CI), IU/L | 16.4 (15.6–17.1) | 16.6 (15.8–17.3) | 18.2 (17.5–19.1) | 19.1 (18.2–19.9) | <0.001 |
| Serum high-sensitivity C-reactive protein, geometric means (95%CI), mg/L | 0.50 (0.44–0.58) | 0.58 (0.51–0.67) | 0.54 (0.47–0.61) | 0.63 (0.55–0.71) | 0.06 |
| Abbreviations: CI, confidence interval; SAM, s-adenosylmethionine; SD, standard deviation.   1. Electrocardiogram abnormalities were defined as left ventricular hypertrophy (Minnesota Code 3-1), ST depression (4-1,2,3), or atrial fibrillation (8-3). 2. Regular exercise was defined as engaging in sports or other forms of exercise at least three times a week during leisure time. | | | | | |

| **Supplementary Table S2. Age- and sex-adjusted baseline characteristics of participants according to serum SAH levels (2007)** | | | | | |
| --- | --- | --- | --- | --- | --- |
| Risk factors | Serum SAH levels (nmol/L) | | | |  |
|  | Q1  (8.96–16.18) | Q2  (16.19–18.95) | Q3  (18.96–23.47) | Q4  (23.48–363.89) | *P* for trend |
|  | (n = 341) | (n = 344) | (n = 343) | (n = 343) |  |
| Age, mean (SD), years | 72 (7) | 73 (6) | 74 (6) | 77 (7) | < 0.001 |
| Men, % | 19.4 | 38 | 49.1 | 67.6 | < 0.001 |
| Education ≤ 9 years, % | 44.2 | 45.6 | 50.6 | 56.5 | 0.002 |
| Systolic blood pressure, mean (SD), mmHg | 133 (19) | 137 (18) | 137 (18) | 137 (19) | 0.01 |
| Diastolic blood pressure, mean (SD), mmHg | 78 (10) | 79 (10) | 80 (10) | 80 (10) | 0.001 |
| Use of anti-hypertensive agents, % | 38.0 | 49.0 | 46.7 | 60.8 | < 0.001 |
| Hypertension, % | 54.4 | 65.9 | 67.8 | 76.5 | < 0.001 |
| Serum total cholesterol, mean (SD), mmol/L | 5.4 (0.9) | 5.3 (0.7) | 5.2 (0.7) | 5.2 (0.9) | 0.001 |
| Use of lipid-modifying agents, % | 24.5 | 25.5 | 20.0 | 23.9 | 0.47 |
| Body mass index, mean (SD), kg/m^2^ | 22.3 (3.5) | 22.6 (3.3) | 23.1 (3.3) | 23.9 (3.5) | < 0.001 |
| Obesity, % | 16.6 | 23.4 | 25.1 | 38.1 | < 0.001 |
| Diabetes mellitus, % | 20.1 | 16.9 | 19.1 | 29.6 | 0.004 |
| Use of antidiabetic medication, % | 9.6 | 7.2 | 10.0 | 18.1 | < 0.001 |
| Electrocardiogram abnormalities, % ^a)^ | 16.7 | 18.0 | 25.9 | 24.1 | 0.01 |
| History of stroke, % | 3.6 | 5.0 | 3.3 | 9.7 | 0.004 |
| Smoking habits, % | 6.3 | 7.6 | 10.3 | 9.2 | 0.12 |
| Alcohol intakes, % | 38.3 | 37.7 | 34.3 | 34.8 | 0.33 |
| Regular exercise, % ^b)^ | 14.6 | 14.2 | 14.9 | 11.1 | 0.28 |
| Use of vitamin B supplements, % | 6.0 | 6.3 | 7.6 | 6.8 | 0.56 |
| Serum alanine transaminase,  geometric means (95%CI), IU/L | 16.3 (15.5–17.1) | 17.1 (16.3–18.0) | 17.3 (16.6–18.2) | 19.3 (18.5–20.5) | <0.001 |
| Serum high-sensitivity C-reactive protein, geometric means (95%CI), mg/L | 0.42 (0.37–0.48) | 0.50 (0.45–0.57) | 0.58 (0.51–0.67) | 0.80 (0.70–0.92) | <0.001 |
| Abbreviations: CI, confidence interval; SAH, s-adenosylhomocysteine; SD, standard deviation.   1. Electrocardiogram abnormalities were defined as left ventricular hypertrophy (Minnesota Code 3-1), ST depression (4-1,2,3), or atrial fibrillation (8-3). 2. Regular exercise was defined as engaging in sports or other forms of exercise at least three times a week during leisure time. | | | | | |

| **Supplementary Table S3. Age- and sex-adjusted baseline characteristics of participants according to serum SAM/SAH ratio levels (2007)** | | | | | |
| --- | --- | --- | --- | --- | --- |
| Risk factors | Serum SAM/SAH ratio levels | | | |  |
|  | Q1  (0.33–2.32) | Q2  (2.33–3.06) | Q3  (3.07–4.52) | Q4  (4.53–15.96) | *P* for trend |
|  | (n = 340) | (n = 343) | (n = 344) | (n = 344) |  |
| Age, mean (SD), years | 76 (7) | 74 (7) | 73 (7) | 73 (7) | < 0.001 |
| Men, % | 57.6 | 46.3 | 35.6 | 34.5 | < 0.001 |
| Education ≤ 9 years, % | 50.7 | 49.3 | 46.6 | 50.3 | 0.77 |
| Systolic blood pressure, mean (SD), mmHg | 135 (18) | 138 (18) | 137 (18) | 135 (18) | 0.71 |
| Diastolic blood pressure, mean (SD), mmHg | 79 (10) | 80 (10) | 80 (10) | 78 (10) | 0.12 |
| Use of anti-hypertensive agents, % | 48.8 | 46.9 | 48.9 | 50.0 | 0.63 |
| Hypertension, % | 65.2 | 66.6 | 69.1 | 64.1 | 0.92 |
| Serum total cholesterol, mean (SD), mmol/L | 5.2 (0.9) | 5.3 (0.7) | 5.2 (0.7) | 5.3 (0.7) | 0.88 |
| Use of lipid-modifying agents, % | 20.9 | 21.4 | 26.9 | 24.6 | 0.12 |
| Body mass index, mean (SD), kg/m^2^ | 22.8 (3.3) | 22.9 (3.3) | 23.1 (3.3) | 23.1 (3.3) | 0.16 |
| Obesity, % | 24.8 | 26.5 | 25.5 | 25.9 | 0.85 |
| Diabetes mellitus, % | 18.5 | 21.1 | 22.0 | 23.9 | 0.09 |
| Use of antidiabetic medication, % | 10.8 | 10.7 | 12.1 | 11.0 | 0.80 |
| Electrocardiogram abnormalities, % ^a)^ | 20.8 | 21.3 | 21.1 | 21.4 | 0.88 |
| History of stroke, % | 7.7 | 4.9 | 4.7 | 4.2 | 0.046 |
| Smoking habits, % | 8.5 | 7.7 | 9.6 | 7.5 | 0.84 |
| Alcohol intakes, % | 30.7 | 40.2 | 37.0 | 37.4 | 0.20 |
| Regular exercise, % ^b)^ | 12.1 | 12.8 | 13.2 | 16.7 | 0.09 |
| Use of vitamin B supplements, % | 5.3 | 7.1 | 8.4 | 5.8 | 0.63 |
| Serum alanine transaminase,  geometric means (95%CI), IU/L | 16.9 (16.3–17.8) | 16.8 (16.0–17.6) | 18.2 (17.3–19.1) | 18.2 (17.3–18.9) | 0.02 |
| Serum high-sensitivity C-reactive protein, geometric means (95%CI), mg/L | 0.61 (0.54–0.70) | 0.58 (0.51–0.66) | 0.53 (0.47–0.61) | 0.53 (0.46–0.60) | 0.09 |
| Abbreviations: CI, confidence interval; SAH, s-adenosylhomocysteine; SAM, s-adenosylmethionine; SD, standard deviation.   1. Electrocardiogram abnormalities were defined as left ventricular hypertrophy (Minnesota Code 3-1), ST depression (4-1,2,3), or atrial fibrillation (8-3). 2. Regular exercise was defined as engaging in sports or other forms of exercise at least three times a week during leisure time. | | | | | |

| **Supplementary Table S4. Risk of all-cause dementia or death according to serum SAM, SAH, and SAM/SAH ratio levels after additionally adjusting for serum alanine transaminase or serum high-sensitivity C-reactive protein (2007**–**2017)** | | | | |
| --- | --- | --- | --- | --- |
|  | Persons  at risk | No. of  events | Hazard ratio (95% CI) | |
|  |  |  | Multivariable-adjusted ^b)^ | Multivariable-adjusted ^c)^ |
| ***Serum SAM*** |  |  |  |  |
| Q1 (10.11–46.39) | 310 | 129 | 1.00 (reference) | 1.00 (reference) |
| Q2 (46.40–60.70) | 316 | 134 | 0.84 (0.67–1.05) | 0.83 (0.66–1.04) |
| Q3 (60.71–88.70) | 317 | 133 | 0.73 (0.58–0.92) ** | 0.75 (0.60–0.94) * |
| Q4 (88.71–579.85) | 320 | 131 | 0.75 (0.60–0.94) * | 0.77 (0.61–0.96) * |
| *P* for trend |  |  | 0.01 | 0.02 |
| Per 1-SD increment ^a)^ |  |  | 0.89 (0.82–0.96) ^$^ | 0.90 (0.83–0.97) ^$^ |
|  |  |  |  |  |
| ***Serum SAH*** |  |  |  |  |
| Q1 (8.96–16.18) | 324 | 110 | 1.00 (reference) | 1.00 (reference) |
| Q2 (16.19–18.95) | 331 | 132 | 0.98 (0.77–1.25) | 0.98 (0.77–1.25) |
| Q3 (18.96–23.47) | 323 | 133 | 0.98 (0.77–1.25) | 0.97 (0.75–1.24) |
| Q4 (23.48–363.89) | 285 | 152 | 1.29 (1.01–1.65) * | 1.27 (0.99–1.62) |
| *P* for trend |  |  | 0.03 | 0.05 |
| Per 1-SD increment ^a)^ |  |  | 1.16 (1.07–1.26) ^$^ | 1.14 (1.05–1.24) |
|  |  |  |  |  |
| ***Serum SAM/SAH ratio*** |  |  |  |  |
| Q1 (0.33–2.32) | 286 | 138 | 1.00 (reference) | 1.00 (reference) |
| Q2 (2.33–3.06) | 328 | 137 | 0.72 (0.58–0.90) ** | 0.73 (0.59–0.92) ** |
| Q3 (3.07–4.52) | 322 | 134 | 0.77 (0.62–0.96) * | 0.80 (0.64–0.99) * |
| Q4 (4.53–15.96) | 327 | 118 | 0.65 (0.52–0.82) ** | 0.67 (0.53–0.84) ** |
| *P* for trend |  |  | < 0.001 | 0.002 |
| Per 1-SD increment ^a)^ |  |  | 0.84 (0.77–0.91) ^$^ | 0.85 (0.78–0.92) ^$^ |
| Abbreviations: CI, confidence interval; PYs, person-years; SAH, s-adenosylhomocysteine; SAM, s-adenosylmethionine; SD, standard deviation.   1. 1-SDs of natural log-transformed values of serum SAM, serum SAH, and the serum SAM/SAH ratio were 0.46, 0.32, and 0.54. 2. Adjusted for age, sex, education, hypertension, diabetes mellitus, serum total cholesterol, body mass index, history of stroke, current smoking, current drinking, regular exercise, use of vitamin B supplements, and serum alanine transaminase (log-transformed). 3. Adjusted for age, sex, education, hypertension, diabetes mellitus, serum total cholesterol, body mass index, history of stroke, current smoking, current drinking, regular exercise, use of vitamin B supplements, and serum high-sensitivity C-reactive protein (log-transformed).   **P* < 0.05, ***P* < 0.01 vs Q1.  ^$^*P* < 0.05 per 1-SD increment. | | | | |

| **Supplementary Table S5. Risk of all-cause dementia or death according to serum SAM, SAH, and SAM/SAH ratio levels after excluding cases occurring within the first 2 years of follow-up (2007**–**2017)** | | | | |
| --- | --- | --- | --- | --- |
|  | Persons  at risk | No. of  events | Hazard ratio (95% CI) | |
|  |  |  | Age- and sex-adjusted | Multivariable-adjusted ^b)^ |
| ***Serum SAM*** |  |  |  |  |
| Q1 (10.11–46.39) | 310 | 129 | 1.00 (reference) | 1.00 (reference) |
| Q2 (46.40–60.70) | 316 | 134 | 0.91 (0.71–1.15) | 0.86 (0.67–1.10) |
| Q3 (60.71–88.70) | 317 | 133 | 0.83 (0.65–1.05) | 0.76 (0.59–0.98) * |
| Q4 (88.71–579.85) | 320 | 131 | 0.80 (0.63–1.03) | 0.76 (0.59–0.97) * |
| *P* for trend |  |  | 0.06 | 0.02 |
| Per 1-SD increment ^a)^ |  |  | 0.91 (0.83–0.99) ^$^ | 0.89 (0.81–0.97) ^$^ |
|  |  |  |  |  |
| ***Serum SAH*** |  |  |  |  |
| Q1 (8.96–16.18) | 324 | 110 | 1.00 (reference) | 1.00 (reference) |
| Q2 (16.19–18.95) | 331 | 132 | 1.09 (0.84–1.40) | 1.05 (0.81–1.36) |
| Q3 (18.96–23.47) | 323 | 133 | 1.06 (0.82–1.37) | 1.01 (0.77–1.31) |
| Q4 (23.48–363.89) | 285 | 152 | 1.29 (0.99–1.67) | 1.16 (0.89–1.52) |
| *P* for trend |  |  | 0.08 | 0.34 |
| Per 1-SD increment ^a)^ |  |  | 1.11 (1.01–1.21) ^$^ | 1.08 (0.98–1.18) |
|  |  |  |  |  |
| ***Serum SAM/SAH ratio*** |  |  |  |  |
| Q1 (0.33–2.32) | 286 | 138 | 1.00 (reference) | 1.00 (reference) |
| Q2 (2.33–3.06) | 328 | 137 | 0.85 (0.67–1.07) | 0.87 (0.68–1.11) |
| Q3 (3.07–4.52) | 322 | 134 | 0.86 (0.68–1.10) | 0.87 (0.68–1.11) |
| Q4 (4.53–15.96) | 327 | 118 | 0.69 (0.54–0.89) ** | 0.72 (0.56–0.93) * |
| *P* for trend |  |  | 0.01 | 0.02 |
| Per 1-SD increment ^a)^ |  |  | 0.86 (0.78–0.95) ^$^ | 0.86 (0.78–0.95) ^$^ |
| Abbreviations: CI, confidence interval; PYs, person-years; SAH, s-adenosylhomocysteine; SAM, s-adenosylmethionine; SD, standard deviation.   1. 1-SDs of natural log-transformed values of serum SAM, serum SAH, and the serum SAM/SAH ratio were 0.46, 0.32, and 0.54. 2. Adjusted for age, sex, education, hypertension, diabetes mellitus, serum total cholesterol, body mass index, history of stroke, current smoking, current drinking, regular exercise and use of vitamin B supplements.   **P* < 0.05, ***P* < 0.01 vs Q1.  ^$^*P* < 0.05 per 1-SD increment. | | | | |

| **Supplementary Table S6. Risk of all-cause dementia or death according to serum methionine, total homocysteine, and methionine/total homocysteine ratio levels (2007**–**2017)** | | | | |
| --- | --- | --- | --- | --- |
|  | Persons  at risk | No. of  events | Hazard ratio (95% CI) | |
|  |  |  | Age- and sex-adjusted | Multivariable-adjusted ^c)^ |
| ***Serum Met*** ^a)^ |  |  |  |  |
| Q1 (13.39–21.66) | 342 | 168 | 1.00 (reference) | 1.00 (reference) |
| Q2 (21.67–24.29) | 343 | 151 | 0.93 (0.74–1.16) | 0.93 (0.74–1.17) |
| Q3 (24.30–27.73) | 343 | 170 | 0.93 (0.75–1.16) | 0.92 (0.74–1.16) |
| Q4 (27.74–58.30) | 343 | 146 | 0.90 (0.70–1.14) | 0.92 (0.72–1.18) |
| *P* for trend |  |  | 0.40 | 0.50 |
| Per 1-SD increment ^b)^ |  |  | 0.95 (0.87–1.04) | 0.97 (0.88–1.06) |
|  |  |  |  |  |
| ***Serum tHcy*** ^a)^ |  |  |  |  |
| Q1 (2.13–7.68) | 340 | 131 | 1.00 (reference) | 1.00 (reference) |
| Q2 (7.69–9.46) | 345 | 143 | 0.86 (0.67–1.09) | 0.91 (0.71–1.16) |
| Q3 (9.47–11.98) | 342 | 146 | 0.87 (0.68–1.11) | 0.89 (0.69–1.14) |
| Q4 (11.99–100.09) | 344 | 215 | 1.33 (1.05–1.67) * | 1.25 (0.98–1.60) |
| *P* for trend |  |  | 0.01 | 0.0495 |
| Per 1-SD increment ^b)^ |  |  | 1.15 (1.06–1.24) ^$^ | 1.14 (1.05–1.24) ^$^ |
|  |  |  |  |  |
| ***Serum Met/tHcy ratio*** |  |  |  |  |
| Q1 (0.17–2.02) | 337 | 207 | 1.00 (reference) | 1.00 (reference) |
| Q2 (2.03–2.57) | 347 | 166 | 0.91 (0.74–1.12) | 0.94 (0.76–1.17) |
| Q3 (2.58–3.23) | 340 | 126 | 0.69 (0.55–0.87) ^$^ | 0.77 (0.61–0.98) * |
| Q4 (3.24–10.95) | 347 | 136 | 0.80 (0.64–1.00) | 0.85 (0.67–1.07) |
| *P* for trend |  |  | 0.01 | 0.06 |
| Per 1-SD increment ^b)^ |  |  | 0.87 (0.81–0.94) ^$^ | 0.88 (0.81–0.95) ^$^ |
| Abbreviations: CI, confidence interval; tHcy, total homocysteine; Met, methionine; PYs, person-years; SD, standard deviation.   1. Units of serum Met and serum tHcy were μmol/L. 2. 1-SDs of natural log-transformed values of serum Met, serum tHcy, and the serum Met/tHcy ratio were 0.19, 0.38, and 0.40. 3. Adjusted for age, sex, education, hypertension, diabetes mellitus, serum total cholesterol, body mass index, history of stroke, current smoking, current drinking, regular exercise and use of vitamin B supplements.   **P* < 0.05, ***P* < 0.01 vs Q1.  ^$^*P* < 0.05 per 1-SD increment. | | | | |

| **Supplementary Table S7. Multivariable-adjusted hazard ratio for the development of Alzheimer's disease or vascular dementia according to serum SAM, SAH, and SAM/SAH ratio levels (2007–2017)** | | | | | | | | |
| --- | --- | --- | --- | --- | --- | --- | --- | --- |
|  | Persons at risk | Alzheimer's disease | | |  | Vascular dementia | | |
|  |  | No. of  events | Crude incidence  (per 1,000 PYs) | Hazard ratio  (95% CI) ^b)^ |  | No. of  events | Crude incidence  (per 1,000 PYs) | Hazard ratio  (95% CI) ^b)^ |
| ***Serum SAM*** |  |  |  |  |  |  |  |  |
| Q1 (10.11–46.39) | 342 | 71 | 26.8 | 1.00 (reference) |  | 18 | 6.8 | 1.00 (reference) |
| Q2 (46.40–60.70) | 343 | 73 | 26.9 | 0.91 (0.66–1.27) |  | 21 | 7.7 | 0.86 (0.45–1.65) |
| Q3 (60.71–88.70) | 343 | 69 | 25.3 | 0.74 (0.53–1.04) |  | 17 | 6.2 | 0.70 (0.35–1.40) |
| Q4 (88.71–579.85) | 343 | 63 | 22.3 | 0.74 (0.52–1.04) |  | 15 | 5.3 | 0.64 (0.32–1.30) |
| *P* for trend |  |  |  | 0.04 |  |  |  | 0.17 |
| Per 1-SD increment ^a)^ |  |  |  | 0.85 (0.75–0.97) ^$^ |  |  |  | 0.84 (0.66–1.07) |
| ***Serum SAH*** |  |  |  |  |  |  |  |  |
| Q1 (8.96–16.18) | 341 | 75 | 25.4 | 1.00 (reference) |  | 9 | 3.0 | 1.00 (reference) |
| Q2 (16.19–18.95) | 344 | 63 | 22.0 | 0.77 (0.54–1.08) |  | 18 | 6.3 | 1.67 (0.75–3.75) |
| Q3 (18.96–23.47) | 343 | 63 | 22.6 | 0.78 (0.55–1.11) |  | 17 | 6.1 | 1.62 (0.71–3.74) |
| Q4 (23.48–363.89) | 343 | 75 | 32.5 | 0.94 (0.66–1.34) |  | 27 | 11.7 | 2.09 (0.92–4.72) |
| *P* for trend |  |  |  | 0.77 |  |  |  | 0.11 |
| Per 1-SD increment ^a)^ |  |  |  | 0.99 (0.86–1.14) |  |  |  | 1.22 (0.97–1.54) |
| ***Serum SAM/SAH ratio*** |  |  |  |  |  |  |  |  |
| Q1 (0.33–2.32) | 340 | 73 | 31.2 | 1.00 (reference) |  | 27 | 11.5 | 1.00 (reference) |
| Q2 (2.33–3.06) | 343 | 73 | 26.0 | 0.84 (0.60–1.18) |  | 8 | 2.8 | 0.30 (0.13–0.67) ** |
| Q3 (3.07–4.52) | 344 | 62 | 22.2 | 0.73 (0.51–1.03) |  | 24 | 8.6 | 0.90 (0.50–1.62) |
| Q4 (4.53–15.96) | 344 | 68 | 23.0 | 0.77 (0.55–1.08) |  | 12 | 4.1 | 0.46 (0.23–0.94) * |
| *P* for trend |  |  |  | 0.09 |  |  |  | 0.20 |
| Per 1-SD increment ^a)^ |  |  |  | 0.88 (0.78–0.99) ^$^ |  |  |  | 0.77 (0.61–0.98) ^$^ |
| Abbreviations: CI, confidence interval; PYs, person-years; SAH, s-adenosylhomocysteine; SAM, s-adenosylmethionine; SD, standard deviation.   1. 1-SDs of natural log-transformed values of serum SAM, serum SAH, and the serum SAM/SAH ratio were 0.46, 0.32, and 0.54. 2. Adjusted for age, sex, education, hypertension, diabetes mellitus, serum total cholesterol, body mass index, history of stroke, current smoking, current drinking, regular exercise and use of vitamin B supplements.   **P* < 0.05, ***P* < 0.01 vs Q1  ^$^*P* < 0.05 per 1-SD increment. | | | | | | | | |

**Supplementary Methods: Quantitation of serum SAM, serum SAH, serum Met, and serum tHcy concentrations**

At the screening examination, portions of the serum specimens were stored at -80°C until used for the measurements of SAM, SAH, Met, and tHcy in 2019 by a validated liquid chromatography-tandem mass spectrometry (LC-MS/MS) method according to the previous study with minor modifications (Guiraud SP, Montoliu I, Silva LD, et al. *Anal Bioanal Chem.* 2017; 409: 295-305). Briefly, after thawing on wet ice, serum (50 μL) was mixed with 10 μL of stable isotope-labeled internal standard (IS) solution (containing 100 nmol/L SAM-d_3_, 10 nmol/L SAH-d_4_, 20 umol/L Met-d_3_, and 8 umol/L Hcys-d_4_) and 50 μL of 50 mg/mL Tris (2-carboxyethyl) phosphine hydrochloride (TCEP-HCl; Tokyo Chemical Industry, Tokyo, Japan), and vortexed for 10 s. After addition of 90 μL of 4% sulfosalicylic acid aqueous solution, the mixture was placed in a multitube vortexer for 30 min at 1000 rpm at 4°C, and further centrifuged at 18,000 × g at 4°C for 10 min. The supernatant was prepared and applied to LC-MS/MS analysis.

An Agilent 6495 Triple Quadrupole LC-MS System equipped with an Agilent 1290 Infinity UHPLC system (Agilent Technologies, Santa Clara, CA) was employed for the analysis. A 1.0 µL aliquot of extracted serum sample solution was injected onto an Agilent Poroshell 120 EC-C18 column (2.7 μm, 2.1 mm i.d. × 100 mm; Agilent Technologies) maintained at 25°C. The mobile phase consisted of solvent A (5 mM perfluoroheptanoic acid (PFHA) in MilliQ water) and solvent B (acetonitrile), and the flow rate was 0.4 mL/min. The elution gradient (A:B, v/v) was adjusted as follows: initial condition 5% B (0–1 min), 5% to 35% B (1–3.5 min), 35% to 40% B (3.5–5.5 min), 40% to 45% B (5.5–7 min), 45% to 95% B (7–7.5 min), 95% B (7.5–9.5 min), and 5% B (10–13 min). Four targeted compounds were analyzed in the positive ion mode, and the detection was carried out in Selected Reaction Monitoring (SRM) mode. Experimental conditions for ESI ionization and mass spectrometry detection are reported in Supplementary Tables S8 and S9. Data were acquired and processed by Agilent MassHunter Workstation Software, version B.08.00. The concentration of analyte in serum was calculated by interpolation of the observed analyte/IS peak-area ratio into the linear regression line for the calibration curve, which was obtained by plotting the peak-area ratios vs analyte concentration using 1/x as the weighting factor. The linear ranges were 10–480 nmol/L (for SAM) , 1–48 nmol/L (for SAH), 2–96 umol/L (for Met) and 1–48 umol/L (for tHcy), and the coefficients of linear correlation (*R^2^*) were >0.99 for the calibration curves of all of the four analytes. Lower limited quantification (LLOQ) was defined as the lowest concentration of the calibration curve with a coefficient of variation (CV) below 20%. Within-batch (n = 100) precision was evaluated by using an in-house pool serum sample, and CV values for the four analytes concentrations in serum were 5.99% (SAM), 6.97% (SAH), 5.72% (Met) and 6.55% (tHcy), respectively. For inter-batch (n = 70) precision evaluation, two in-house pool serum samples were used for analysis before and after the batch samples analysis in addition to the QC samples, and their CV values for the four analytes concentrations were 8.97% and 9.53% (SAM), 10.23% and 12.45% (SAH), 6.27% and 7.38% (Met), and 6.34% and 8.30% (tHcy), respectively. Accuracy was assessed by spiking in-house pool serum samples at three different concentrations of four analytes standards in six replicates for each level (Supplementary Table S10).

**(Chemicals and reagents)**

S-Adenosylmethionine (Ademetionine, SAM) was purchased from SCB Santa Cruz Biotechnology Inc. (Dallas, TX). S-(5'-Adenosyl)-L-methionine-d_3_ (SAM-d_3_) and DL-Homocysteine-d_4_ (Hcys-d_4_) were purchased from Toronto Research Chemicals Inc. (Toronto, Canada). S-(5'-Adenosyl)-L-homocysteine (SAH), L-homocysteine (Hcys), L-methionine-(methyl-d_3_) (Met-d_3_) and perfluoroheptanoic acid (PFHA) were purchased from Sigma-Aldrich (St. Louis, MO). S-Adenosyl-L-Homocysteine-d_4_ (SAH-d_4_) was purchased from Cayman Chemical Co. (Ann Arbor, MI). L-Methionine was purchased from Wako Pure Chemical Industries, Ltd. (Osaka, Japan). Tris (2-carboxyethyl) phosphine hydrochloride (TCEP-HCl) was purchased from Tokyo Chemical Industry (Tokyo, Japan). LC-MS-grade acetonitrile (ACN) was purchased from Wako Pure Chemical (Osaka, Japan), and ultrapure water was produced by a Millipore-Q water system (Millipore, Bedford, MA).

| **Supplementary Table S8. Conditions for ESI ionization** | |
| --- | --- |
| Parameter | Value (+) |
| Gas Temp (°C) | 200 |
| Gas Flow (l/min) | 15 |
| Nebulizer (psi) | 20 |
| Sheath Gas Heater | 350 |
| Sheath Gas Flow | 12 |
| Capillary (V) | 3500 |

| **Supplementary Table S9. Selected reaction monitoring transitions for each compound** |
| --- |

| Compound | MRM transition (*m*/*z*) | Collision energy (eV) | Retention time  (min) |
| --- | --- | --- | --- |
| SAM | 399.2 > 250.2 | 13 | 5.91 |
| SAM-d_3_ | 402.2 > 250.1 | 17 | 5.91 |
| SAH | 385.1 > 136.1 | 29 | 5.84 |
| SAH-d_4_ | 389.2 > 138.0 | 21 | 5.84 |
| Met | 150.1 > 104.0 | 10 | 4.10 |
| Met-d_3_ | 153.1 > 107.1 | 9 | 4.10 |
| Hcys | 136.0 > 90.0 | 10 | 3.30 |
| Hcys-d_4_ | 140.1 > 94.1 | 9 | 3.30 |
| Abbreviations: SAM, s-adenosylmethionine; SAH, s-adenosylhomocysteine; Met, methionine; Hcys, homocysteine. | | | |

| **Supplementary Table S10. Accuracy data for the LC-MS/MS method** | | | | |
| --- | --- | --- | --- | --- |
| Compound | Concentration ^a)^ | | Recovery (%) | CV (%) |
|  | Serum Original | Spiked |  |  |
| SAM | 157.4 | 40 | 100.7 | 12.2 |
|  | 157.4 | 80 | 85.7 | 13.7 |
|  | 157.4 | 120 | 93.1 | 6.1 |
| SAH | 5.8 | 4 | 96.3 | 17.2 |
|  | 5.8 | 8 | 103.4 | 9.1 |
|  | 5.8 | 12 | 98.6 | 5.4 |
| Met | 20.7 | 8 | 88.9 | 4.6 |
|  | 20.7 | 16 | 102.0 | 5.4 |
|  | 20.7 | 24 | 96.5 | 4.4 |
| tHcy | 11.0 | 4 | 105.1 | 10.7 |
|  | 11.0 | 8 | 109.5 | 1.1 |
|  | 11.0 | 12 | 109.2 | 2.0 |
| Abbreviations: SAH, s-adenosylhomocysteine; SAM, s-adenosylmethionine; Met, methionine; tHcy, total homocysteine.   1. Units of serum SAM and serum SAH were nmol/L and serum Met and serum tHcy were μmol/L. | | | | |


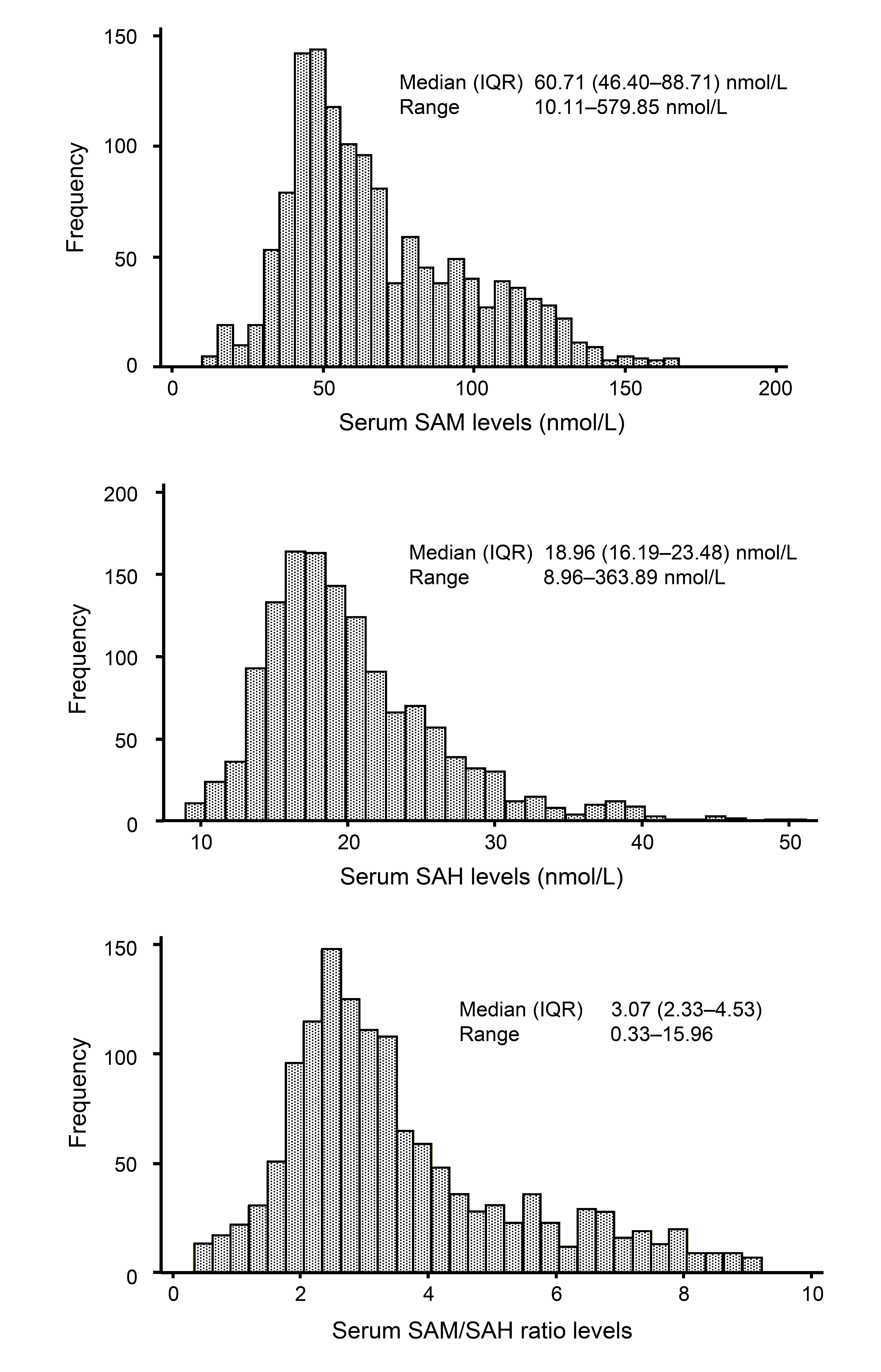


**Supplementary Figure S1. Histogram of serum SAM, SAH, and SAM/SAH ratio**

Histogram of serum SAM, SAH, and SAM/SAH ratio levels in the study population (n = 1,371), 2007. Values above the 99th percentile are not shown in the figure.

SAM, s-adenosylmethionine; SAH, s-adenosylhomocysteine.
